# Supplementary material for: WordCommentsAnalyzer: A windows software tool for qualitative research
Source: F1000Res. 2018 Sep 4;7:536. Originally published 2018 May 3. [Version 2] doi: 10.12688/f1000research.14819.2 (PMC6137411; doi:10.12688/f1000research.14819.2)
Supplement: Supplementary file 3 [file f1000research-7-17614-s0002.tgz › b2a79496-4ada-47c4-9682-a99ce867836d.zip!/Eight Iranian Health Journals/Archives of Rehabilitation_33.docx]

© Archives of Rehabilitation. This work is licensed under CC BY-NC 4.0 (https://creativecommons.org/licenses/by-nc/4.0/).

Volume & Issue: دوره 12 - شماره 2

Title: تأثیر توانبخشی قلبی بر اضطراب و افسردگی بیماران تحت عمل جراحی پیوند عروق کرونر در بیمارستانهای وابسته به دانشگاه علوم پزشکی شیراز

http://rehabilitationj.uswr.ac.ir/article-1-385-fa.html

هدف: بسیاری از بیماران پس از عمل جراحی قلب به‌علت استرس پس از جراحی، با اضطراب و افسردگی مواجه می‌گردند. این پژوهش با هدف تعیین تأثیر توانبخشی قلبی بر اضطراب و افسردگی بیماران پس از پیوند عروق کرونر صورت گرفته‌ است. روش‌بررسی: در این مطالعه مداخله‌ای و تجربی، 80 نفر از بیماران جراحی‌شده پیوند عروق کرونر در بیمارستان‌های وابسته به دانشگاه علوم پزشکی شیراز که دارای ویژگیهای شرکت در مطالعه بودند، بر اساس روش نمونه‌گیری مبتنی بر هدف انتخاب و به‌روش تصادفی به دو گروه آزمایش و شاهد تخصیص یافتند. ابزار گردآوری داده‌ها شامل پرسشنامه اطلاعات جمعیت شناختی، پرسشنامه اضطراب اسپیلبرگر و پرسشنامه افسردگی بک بود. پژوهش طی سه مرحله انجام شد: ابتدا در زمان ترخیص میزان اضطراب و افسردگی در دو گروه شاهد و آزمایش مورد سنجش قرار گرفت. در مرحله دوم برای گروه آزمایش به‌مدت 4 هفته توانبخشی قلبی (شامل تمرینات ورزشی، رژیم دارویی و غذایی،کنترل وزن و استرس و آرامسازی) به‌صورت گروههای 5 نفره آموزش داده شده و پیگیری شد. گروه شاهد فقط مراقبت‌های معمول را دریافت می‌کرد. در پایان توانبخشی قلبی اضطراب و افسردگی بیماران دو گروه اندازه‌گیری شد و در مرحله سوم (2 ماه پس از اتمام توانبخشی قلبی) مجدداً اضطراب و افسردگی بیماران مورد سنجش قرار گرفت. داده‌های جمع‌آوری شده با استفاده از آزمون‌های مجذور کای و اندازه‌های تکراری چند متغیره مورد تجزیه و تحلیل قرار گرفت. یافته‌ها: بعد از اجرای توانبخشی قلبی تفاوت آماری معناداری از نظر میزان افسردگی میان دو گروه دیده شد(0/014=P)، اما در زمینه اضطراب تفاوت آماری معناداری بین دو گروه دیده نشد (0/079=P). نتیجه‌گیری: توانبخشی قلبی در کاهش افسردگی بیماران پس از جراحی پیوند عروق کرونر مؤثر می‌باشد.

Title: بررسی وضعیت بهداشت دندان در بیماران اسکیزوفرنیک بستری در مراکز مراقبت از بیماران روانی مزمن استان چهارمحال و بختیاری در سال 1387

http://rehabilitationj.uswr.ac.ir/article-1-343-fa.html

هدف: بیماران اسکیزوفرنیک در انجام امور شخصی و از جمله بهداشت دهان و دندان ناتوان بوده و اثرات جانبی داروهای مصرفی همچون داروهای ضدروانپریشی باعث بروز مشکلات دهانی در آنها می‌ ‌‌ شود. این مطالعه با هدف تعیین وضعیت بهداشت دندان در بیماران اسکیزوفرنیک بستری در مراکز مراقبت از بیماران روانی مزمن استان چهارمحال و بختیاری انجام شده ‌‌ است.  روش بررسی: این مطالعه توصیفی-تحلیلی و مقطعی در سال 1387 بر روی 123 بیمار اسکیزوفرنیک بستری در سه مرکز نگهداری بیماران روانی مزمن استان چهارمحال و بختیاری با نمونه ‌ گیری به شکل تمام‌شمار صورت گرفت. جمع‌آوری اطلاعات با ‌‌ استفاده از پرسشنامه استاندارد علائم مثبت و منفی آندرسون – از طریق مصاحبه روانپزشکی – پرسشنامه اطلاعات دموگرافیک، ایندکس لثه ‌ ای و فرم اطلاعات مربوط به وضعیت دندانها صورت گرفت. از آزمون‌های آماری مان‌ویتنی‌‌، خی2 و محاسبه ضریب همبستگی اسپیرمن برای تجزیه و تحلیل داده‌‌ها استفاده شد. یافته‌ها: میانگین مربوط به تعداد دندان ‌ های کشیده شده، پُرشده و لازم به ترمیم در جمعیت مورد مطالعه ۷٫۷۱±۱۹٫۴۳ بوده و رابطه معناداری بین این میانگین و(۰/527=r؛0/001>P)، سابقه مصرف سیگار (۰/۳۳۲=r؛۰/۰۰۱>P)، تعداد نخ سیگار مصرفی در روز (۰/۱۷۱=r؛۰/۰۳۱>P) ، مدت زمان بستری فعلی (0.157=r؛ 0.043>P) و میانگین نمرات علائم منفی وجود داشت. از نظر وضعیت لثه، شدت پیوره در مردان به شکل معناداری شدیدتر از زنان بود (۰/۰۰۱> P) و سابقه مصرف سیگار، تعداد نخ سیگار مصرفی در روز، سابقه بستری در بیمارستان و میانگین نمرات علائم مثبت و منفی نیز با شدت پیوره رابطه آماری معناداری نشان داد ( ۰٫۰۵> P) .  نتیجه‌گیری: یافته ‌‌ های این مطالعه نشان می‌ ‌‌ دهد وضعیت بهداشت دهان و دندان بیماران اسکیزوفرنیک نامناسب می‌ ‌‌ باشد، بنابراین لزوم ارائه مراقبت ‌‌ های بهداشتی و به ‌‌ ویژه بهداشت دهان و دندان در کنار سایر درمان ‌‌ ها و مراقبت ‌‌ های معمول مطرح می‌ ‌‌ باشد.

Title: بررسی تأثیر روش درمانی تعدیل‌شده معکوس‌سازی عادات در درمان لکنت زبان در خانه ، مدرسه و کلینیک

http://rehabilitationj.uswr.ac.ir/article-1-316-fa.html

هدف: لکنت زبان اختلالی است که می‌تواند سبب ایجاد مشکلات فردی، عاطفی و اجتماعی زیادی شود. بر مبنای پاتوفیزیولوژی‌های مطرح شده برای لکنت، در مورد درمان لکنت نظرات متعددی وجود دارد. در این بین درمان‌های رفتاری از اثرات مثبتی در درمان لکنت برخوردار بوده‌اند. هدف این بررسی تأثیر روش درمانی تعدیل‌شده معکوس‌سازی عادات ( Habit-reversal Simplified ) به‌عنوان یک روش رفتاری در درمان لکنت زبان در کودکان ایرانی است. روش‌بررسی: 12 دانش‌آموز از تعداد 350 دانش‌آموز دوره دبستان و راهنمایی شهر کرمان که مبتلا به لکنت بودند، در این بررسی شبه‌تجربی شرکت کردند. پس از ارزیابی اولیه در مورد شدت لکنت کودکان در خانه، مدرسه و کلینیک روش درمانی تعدیل‌شده معکوس‌سازی عادات برای این افراد انجام شد. جلسات پی‌گیری ارزیابی در سه محیط خانه، مدرسه و کلینیک در ماه‌های ۱۲،۶،۳،۲،۱ و ۱۳پس از درمان انجام شد. از آزمون‌های آماری اندازه‌های تکراری و آزمون تعقیبی آزمون تی زوج جهت آنالیز آماری استفاده شد.  یافته‌ها: میزان لکنت در ارزیابی‌های مختلف در محیط خانه نسبت به ارزیابی پایه، کاهش معنادار نشان داد(۰/۳۳ >P). میزان لکنت در ارزیابی‌های مختلف در محیط مدرسه (۰/۰۲۵>P) و کلینیک(۰/۰۳۵>P) نسبت به ارزیابی پایه نیز، کاهش معنادار نشان داد . تغییرات لکنت در محیط‌های مختلف رابطه معناداری با یکدیگر نداشتند(۰/۷۳=P). کاهش لکنت با متغیر‌های دموگرافیک ارتباطی نداشت.  نتیجه‌گیری: به‌نظر می‌رسد روش تعدیل‌شده معکوس‌سازی عادات در درمان لکنت زبان در کودکان ایرانی و در تمام محیط‌های خانه، مدرسه و کلینیک مؤثر می‌باشد، اگرچه مطالعات بیشتر در موقعیت‌های مختلف درمانی و نیز سایر اختلالات روانی همراه توصیه می‌شود.

Title: تأثیر تحریک حسی با استفاده از جلیقه وزین بر مهارت‌های حرکتی و توجه در کودکان مبتلا به اختلالات یادگیری

http://rehabilitationj.uswr.ac.ir/article-1-906-fa.html

هدف: رشد توجه و مهارتهای حرکتی در یادگیری، رشد و کارهای روزمره زندگی کودکان مبتلا به اختلالات یادگیری اهمیت زیادی دارد. این در حالی است که نقص قابل توجهی در زمینه مهارتهای حرکتی و توجه در این کودکان مشاهده می‌شود. هدف پژوهش حاضر بررسی تأثیر تحریک حسی به‌وسیله جلیقه وزین بر مهارتهای حرکتی و توجه کودکان 10-6 ساله دارای اختلالات یادگیری بود. روش‌بررسی: در این مطالعه مداخله‌ای و تجربی، شرکت‌کنندگان در پژوهش شامل 12 کودک 10-6 ساله مبتلا به اختلالات یادگیری مراجعه کننده به مراکز توانبخشی وابسته به دانشگاه علوم بهزیستی و توانبخشی بودند که به‌صورت تصادفی در دو گروه مداخله و مقایسه جای گرفتند. نمونه‌ها از طریق نمونه‌گیری ساده و در دسترس انتخاب شدند. مهارتهای حرکتی و توجه طی دو مرحله پیش‌آزمون و پس‌آزمون با استفاده از مقیاس رشد حرکتی لینکلن ازرتسکی و مقیاس رتبه‌بندی کانرز اندازه‌گیری و نتایج ثبت شد. مداخله پژوهش به‌صورت ارائه تحریک حسی مداوم با استفاده از جلیقه وزین در طی خدمات معمول کاردرمانی به قرار 3 جلسه 45 دقیقه‌ای در هفته و برای 12 جلسه بر روی گروه مداخله اجرا شد. داده‌های پژوهش با استفاده از آزمون رتبه‌های نشان دار ویلکاکسون و آزمون من- ویتنی تحلیل شد. یافته‌ها: بین نمرات پیش‌آزمون و پس‌آزمون مهارت‌های حرکتی در گروه مداخله تفاوت معناداری وجود داشت(۰/۰۰۳=P)، ولی در گروه مقایسه تفاوت معناداری وجود نداشت. همچنین بین نمرات پیش‌آزمون و پس‌آزمون توجه در گرو‌ههای مداخله و مقایسه تفاوت معناداری وجود نداشت.  نتیجه‌گیری: یافته‌های این پژوهش حاکی از آن است که تحریک حسی مبتنی بر جلیقه وزین از لحاظ آماری بر مهارت‌های حرکتی مؤثر بوده، اما بر توجه تأثیر معناداری ندارد؛ لذا می‌توان از این فن در جهت بهبود رشد مهارت‌های رشد حرکتی در کودکان مبتلا به اختلالات یادگیری بهره گرفت.

Title: نامه به سردبیر: آیا تزریق سلول‌های بنیادی در درمان کودکان فلج مغزی مؤثر است؟

http://rehabilitationj.uswr.ac.ir/article-1-838-fa.html

فلج مغزی گروهی از اختلالات مغزی است که شایع‌ترین ناتوانی حرکتی مزمن را به دنبال دارد. مهم‌ترین مداخلات درمانی بر روی این گروه را توانبخشی، دارو درمانی و اقدامات جراحی تشکیل می‌دهند. هدف نهایی این مداخلات، کاهش علایم و عوارض این اختلال، در مغز در حال رشد است که به صورت بهبودی در کنترل حرکات و هماهنگی عضلات قابل مشاهده خواهد بود. عوارضی نظیر اسپاستیسیتی، ضعف عضلانی، اندازه طولی کمتر عضلات نسبت به استخوان‌ها و … زمینه را جهت بروز اختلالات ثانویه فراهم می‌آورد. تاکنون هیچ مداخله بالینی قادر به ترمیم جبرانی یا نوروپلاستیسیته در سلول‌های آسیب دیده مغزی، وجود نداشته است. به طور کلی سه مسیر تحقیقاتی در خصوص ترمیم مغز آسیب دیده وجود دارد:   (1) جایگزین نمودن سلول‌های مغزی که آسیب دیده یا مرده‌اند یا عملکرد بسیار ضعیفی دارند.   (2) ترمیم توالی‌های عصبی آسیب دیده مثل دندریت‌ها و آکسون‌ها.   (3) تحریک مسیرهای مغزی جایگزین جهت بهبود حرکت و هماهنگی.   امروزه مراکز تحقیقاتی فعال در زمینه سلول‌های بنیادی (سلول‌هایی که قابلیت تکثیر و یافتن محل آسیب دیده را دارند) تحقیقات خود را برپایه مسیرهای 1 و 2 از سه روش فوق قرار داده‌اند. مراکز تحقیقاتی خارج از کشور گزارشاتی حاکی از مؤثر بودن این روش و آمارهای بسیار خیره‌کننده از موفقیت آن می‌دهند؛ البته موفقیت در سنین زیر ۵ سال بالاتر گزارش شده است. به عنوان مثال مرکز تحقیقاتی ایکس سل اعلام کرده است که احتمال موفقیت و بهبودی هفتاد درصد است. با توجه به مزمن بودن اختلال و مشکلات عدیده‌ای آن برای خانواده و شخص مبتلا از لحاظ مالی، روانی، عاطفی و...، چنانچه این روش بتواند ادعاهای خود را ثابت نماید می‌توان گفت علی‌رغم هزینه‌های سنگین مالی (به عنوان مثال هزینه انجام این تزریق به همراه هزینه‌های جانبی مراجعه به مرکز ایکس سل در آلمان، حدود 16 میلیون تومان تخمین زده می‌شود، قابل پیشنهاد به طیفی از مراجعین کلینیکی خواهد بود.   از این رو در ادامه گزارشی از ارزیابی کلینیکی یک کودک فلج مغزی اسپاستیک قبل و بعد از تزریق سلول‌های بنیادی و مقایسه آن ارائه می‌گردد.   کودک، 9 ساله ساکن تهران و مبتلا به فلج اسپاستیک چهار اندام بود، ارزیابی‌ها قبل از درمان شامل وجود اسپاستیسیتی درجه 3 در سراسر اندام تحتانی بر اساس مقیاس آشورت و وجود اختلالات بینایی نظیر نیستاگموس را نشان می‌داد. از بُعد حرکات درشت، تنها قادر به غلتیدن و نشستن با پاهای باز بود. میزان اسپاستیسیتی در اندام فوقانی سمت راست بیشتر از سمت چپ بوده و همچنین کودک سابقه جراحی در اندام تحتانی را نیز داشت. در مرکز ایکس سل در روز اول مغز استخوان از طریق خار خاصره استخوان لگن با بیهوشی عمومی به دست آورده شد و در روز بعد در آزمایشگاه به کشت رسید. در روز سوم سلول‌های بنیادی از طریق مایع مغزی نخاعی کمر تزریق گردید. پس از آن بیمار مرخص شد؛ لکن باید در نزدیکی مرکز تا اطمینان از سلامت عمومی حضور می‌داشت. پس از طی دوره 15 روزه بیمار به کشور بازگشت.   در اولین جلسه‌ای که بیمار به کلینیک توانبخشی مراجعه نمود، نتایج ارزیابی‌های کلینیکی به شرح زیر به دست آمد: اسپاستیسیتی در سراسر اندام تحتانی از درجه 3 به درجه 2 بر اساس مقیاس آشورث کاهش یافته بود، حرکات چشم (نیستاگموس) کاهش یافته و تا حدودی بهبودی در ثبات وضعیت تنه قابل مشاهده بود. در سایر موارد بهبودی مشاهده نگردید.   با توجه به عدم تغییر معنادار در وضعیت مراجع، درمان‌های متداول توانبخشی بر روی او همچنان ادامه دارد و مراجع می‌بایست شش ماه دیگر جهت ارزیابی مجدد به مرکز ایکس سل مراجعه نماید. البته با توجه به اینکه کودک فوق الذکر ۹ ساله بوده و در این سن قاعدتاً دفورمیتی‌ها و کانترکچرها دیگر ثابت و پایدار می‌شوند انتظار اینکه چنین بیماری حتی در صورت ترمیم سلول‌ها آسیب‌دیده مغزی‌اش علائم ظاهری‌اش بهبود یابند، دور از ذهن به‌نظر می‌رسد. از سوی دیگر سیناپتوژنز در مغز نیز دیگر در این سن بسیار کند می‌شود و علاوه بر آن به دلیل عدم تحرک کافی سیناپتوژنزهای برقرار شده ممکن است تحلیل رفته و نتواند عملکرد واقعی خود را نشان دهد. بنابراین انجام تحقیق تجربی در گروه‌های سنی پایین‌تر و نیز بررسی وضعیت میزان بهبود افراد با سن بالاتر جهت روشن شدن موضوع بسیار مفید خواهد بود. از این رو از همکاران محترم درخواست می‌شود چنانچه اطلاعاتی در این خصوص داشته و تمایل به همکاری پژوهشی هستند با نویسنده مقاله و یا مرکز تحقیقات توانبخشی اعصاب اطفال دانشگاه علوم بهزیستی و توانبخشی تماس حاصل نماید.

Title: ارزیابی میزان فشار کف ‌پایی در بیماران دیابتی نوع ۲ دارای نوروپاتی خفیف

http://rehabilitationj.uswr.ac.ir/article-1-671-fa.html

هدف: بیماران مبتلا به دیابت نوروپاتیک، فاقد حس درد در کف ‌پای خود بوده و در معرض ایجاد زخمهای فشاری می‌باشند. هدف این مطالعه اندازه‌گیری فشار کف‌‌ِپایی بیماران دیابتی نوع ۲ دارای نوروپاتی خفیف و مقایسه آن با افراد سالم و همچنین تعیین نقاط در معرض خطر ایجاد زخم در کف پای این بیماران می‌باشد. روش بررسی: در این مطالعه مقطعی-مقایسه‌ای، میزان فشار کف‌‌پایی بیست نفر شرکت‌کننده انتخاب شده با روش نمونه‌گیری قضاوتی که در دو گروه ۱۰ نفره افراد سالم و بیماران دیابتی قرار می‌گرفتند، در هنگام راه‌رفتن توسط دستگاه اسکن پا در ده ناحیه کف پا مورد ارزیابی قرار گرفت. داده‌های حاصل با استفاده از آزمون آماری تی مستقل تحلیل شد. یافته‌ها: حداکثر میزان فشار کف‌‌پایی در زیر سرِ استخوانهای کف‌‌پایی (متاتارس) ۲ و ۴ و حداقل آن در زیر انگشتان ۲ تا ۵ قرار داشت. اگرچه در هشت ناحیه کف پا میزان فشار کف‌‌پایی در بیماران دیابتی نسبت به افراد سالم بیشتر بود، اما مقدار اختلاف میانگین دو گروه از لحاظ آماری معنادار نبود. نتیجه‌گیری: عدم اختلاف معنادار در میزان فشار کف‌ِپایی بیماران دیابتی در مقایسه با افراد سالم، احتمالاً به‌دلیل خفیف بودن شدت نوروپاتی و عدم وجود زخم در کف ‌پای این بیماران بوده ‌است. همچنین سر متاتارس 3 و 4 این بیماران بیشتر مستعد ایجاد زخم بود.

Title: بررسی مقایسه‌ای نقش ابعاد جهت‌گیری مذهبی و ابعاد شخصیت در پیش‌بینی سبک‌های مقابله مادران کودکان با و بدون ناتوانی ذهنی

http://rehabilitationj.uswr.ac.ir/article-1-581-fa.html

هدف: هدف از پژوهش حاضر بررسی مقایسه‌ای نقش ابعاد شخصیت و ابعاد جهت‌گیری مذهبی در پیش‌بینی سبک‌های مقابله با استرس در مادران با و بدون کودک دارای ناتوانی ذهنی بود. روش: در این مطالعه توصیفی از نوع تحلیل همبستگی، 124 مادر دارای کودک ناتوان ذهنی و 124 مادر دارای کودک بدون ناتوانی ذهنی به صورت نمونه گیری تصادفی خوشه‌ای از بین مراکز آموزشی منطقه 5 و 6 تهران انتخاب شدند. ابزار پژوهش شامل پرسشنامه‌های مذهب‌گرایی اسلامی، پنج عامل شخصیت و سبک‌های مقابله بود و از آزمون‌های آماری تحلیل همبستگی و رگرسیون برای تحلیل داده‌ها استفاده شد. یافته‌ها: مقایسه دو گروه نشان داد که باورهای مذهبی خصوصاً مذهب‌گرایی و ‌ارزنده‌سازی مذهبی نقش مهمی در پیش‌بینی سبک مقابله مسئله‌محور در مادران دارای کودک ناتوان ذهنی نسبت به مادران دارای کودک بدون ناتوانی ذهنی دارند، و از بین ابعاد شخصیت، بعد روان‌نژندی نقش بیشتری در پیش‌بینی سبک‌های مقابله هیجان‌محور در مادران دارای کودک ناتوان ذهنی دارد (۰/۰19=P) . اما باورهای مذهبی در مادران دارای کودک ناتوان ذهنی در قیاس با مادران دارای کودک بدون ناتوانی ذهنی نقش معناداری در پیش‌بینی سبک مقابله هیجان‌محور نداشت. نتیجه گیری: در قیاس با مادران دارای کودک بدون ناتوانی ذهنی، باورهای مذهبی پیش‌بینی‌کننده‌های خوبی برای سبک مقابله مسئله‌محور و ابعاد شخصیت (عمدتاً روان‌نژندی) پیش‌بینی‌کننده‌های خوبی برای سبک مقابله هیجان‌محور در مادران دارای کودک ناتوان ذهنی هستند.

Title: تفاوتهای معناشناختی مهارت تعریف واژه در کودکان فارسی زبان دارای اختلال ویژه زبان و طبیعی

http://rehabilitationj.uswr.ac.ir/article-1-886-fa.html

هدف: مهارت تعریف واژه یکی از مهارتهای پیچیده زبان است که دانش زبانی و فرازبانی نقش موثری در آن دارند. در این مطالعه مهارت تعریف واژگان اسمی کودکان مبتلا به اختلال ویژه زبان (SLI) به لحاظ محتوایی (معنایی)وساختاری مورد بررسی و مقایسه با کودکان طبیعی قرار گرفت. روش بررسی: این پژوهش از نوع مقطعی توصیفی- تحلیلی می باشد که در طی آن 32 دانش آموز انتخاب شده و مورد بررسی قرار گرفتند. 16 دانش آموز مبتلا به اختلال ویژه زبان مراجعه کننده به مراکز مشکلات یادگیری و مرکز توانبخشی شهید زارعی و 16 دانش آموز طبیعی نیز بصورت تصادفی انتخاب شده واز نظر سن، جنس و مقطع تحصیلی با گروه آزمایش همتا سازی شدند. سپس آزمون محقق ساخته تعریف واژه اجرا گردید و نتایج با استفاده از روش آماری تی- تست مورد مقایسه قرار گرفتند. پایایی این آزمون به روش همبستگی نمرات دو ارزشیاب مستقل محاسبه و روایی آن به صورت محتوایی ارزشیابی شد. یافته ها: گروه طبیعی میانگین امتیازات بالاتری نسبت به گروه دارای اختلال در هر دو جنبه محتوایی و ساختاری تعریف واژه کسب کردند بطوریکه میانگین و انحراف معیار امتیاز جنبه محتوایی تعریف واژه در گروه طبیعی M= 45/87, SD=12/22 و در گروه مبتلا (17/60=M=33/18, SD) از 70 امتیازبود و تفاوت دو گروه معنی دار بود (P= 0/025). میانگین و انحراف معیار امتیازجنبه ساختاری تعریف واژه در گروه طبیعی (M= 48/87, SD= 9/49) و در گروه مبتلا M= 38/18, SD= 12/85 از 70 امتیاز بود و دو گروه دارای تفاوت معنی دار بودند (P= 0/012). نتیجه گیری: با توجه به یافته های پژوهش می توان متوجه شد که مشکلات زبانی کودکان SLI اجازه بازنمایی معنایی جهت تشکیل و ارائه تعریفی جامع از واژه را نمی دهد. با وجودیکه مهارت تعریف واژه در کودکان SLI ضعیف است، با طبقه بندی جنبه محتوایی و ساختاری تعریف واژگان اسمی مورد استفاده دراین مطالعه همخوانی دارد.بنابراین امکان تقویت این مهارت با مداخله درمانگران گفتار و زبان بویژه در بخش معناشناختی و ساختاری وجود دارد. این مداخله ممکن است علاوه بر تقویت تعریف مفاهیم آشنا به رشد تعریف مفاهیم جدید نیز کمک کند که نتیجه آن پیشرفت تحصیلی وارتباطی این دسته از کودکان می باشد.

Title: مقایسه استقامت عضلات کمربند شانه‌ای در بیماران مبتلا به سندرُم گیرافتادگی و افراد سالم

http://rehabilitationj.uswr.ac.ir/article-1-965-fa.html

هدف: تغییرات خفیف در عملکرد و هماهنگی عضلات کتف و گلنوهومرال می‌تواند منتهی به اختلال مفصل شانه گردد. سندرُم گیرافتادگی به‌عنوان شایعترین علت درد شانه گزارش شده است. هدف از این مطالعه بررسی استقامت عضلات مجموعه شانه دربیماران مبتلا به سندرُم گیرافتادگی بوده است.  روش بررسی: با استفاده از نمونه‌گیری غیراحتمالی ساده 15 بیمار مبتلا به سندرُم گیرافتادگی شانه با میانگین سنی ۴۵٫۳۰ سال و 15 فرد سالم (سن ۴۵/۸۰ سال) طی یک مطالعه موردی-شاهدی مورد مقایسه قرار گرفتند. استقامت عضلات گلنوهومرال و اسکاپولوتوراسیک با استفاده از یک دینامومتر دستی مورد بررسی قرار گرفت. از آنالیز آماری تی مستقل برای مقایسه گروهها استفاده گردید. یافته‌‌ها: در مقایسه با افراد سالم بیماران مبتلا به سندرُم گیرافتادگی دارای استقامت کمتری در عضلات مسئول حرکات چرخش به خارج(۰/۰۲=P)، اسکاپشن (۰/۰۰۱>P) و عضلات چرخاننده کتف به بالا بودند(۰/۰۵=P). در بیماران مبتلا به سندرُم گیرافتادگی نسبت استقامت عضلات چرخاننده به خارج به استقامت عضلات چرخاننده به داخل به‌طور معناداری کمتر از گروه کنترل بود(۰/۰۰۱> P). نتیجه‌‌گیری: نتایج این مطالعه کاهش استقامت عضلات روتیتور کاف و چرخاننده‌‌های به سمت بالای کتف را در بیماران مبتلا به سندرُم گیرافتادگی نشان می‌دهد. بنابراین استقامت عضلانی باید در برنامه ارزیابی و درمان فیزیوتراپی این بیماران در نظر گرفته شود.
